# Screening the Global Health Priority Box against *Plasmodium berghei* liver stage parasites using an inexpensive luciferase detection protocol

**DOI:** 10.1186/s12936-024-05155-y

**Published:** 2024-11-23

**Authors:** Gia-Bao Nguyen, Caitlin A. Cooper, Olivia McWhorter, Ritu Sharma, Anne Elliot, Anthony Ruberto, Rafael Freitas, Ashutosh K. Pathak, Dennis E. Kyle, Steven P. Maher

**Affiliations:** Center for Tropical and Emerging Global Diseases, University of Georgia, 500 DW Brooks Dr, Athens, GA 30602 USA

## Abstract

**Background:**

Malaria, a disease caused by parasites of the genus *Plasmodium*, continues to impact many regions globally. The rise in resistance to artemisinin-based anti-malarial drugs highlights the need for new treatments. Ideally, new anti-malarials will kill the asymptomatic liver stages as well as the symptomatic blood stages. While blood stage screening assays are routine and efficient, liver stage screening assays are more complex and costly. To decrease the cost of liver stage screening, a previously reported luciferase detection protocol requiring only common laboratory reagents was adapted for testing against luciferase-expressing *Plasmodium berghei* liver stage parasites.

**Methods:**

After optimizing cell lysis conditions, the concentration of reagents, and the density of host hepatocytes (HepG2), the protocol was validated with 28 legacy anti-malarials to show this simple protocol produces a stable signal useful for obtaining quality small molecule potency data similar to that obtained from a high content imaging endpoint. The protocol was then used to screen the Global Health Priority Box (GHPB) and confirm the potency of hits in dose–response assays. Selectivity was determined using a galactose-based, 72 h HepG2 assay to avoid missing mitochondrial-toxic compounds due to the Crabtree effect. Receiver-operator characteristic plots were used to retroactively characterize the screens’ predictive value.

**Results:**

Optimal luciferase signal was achieved using a lower HepG2 seed density (5 × 10^3^ cells/well of a 384-well microtitre plate) compared to many previously reported luciferase-based screens. While producing lower signal compared to a commercial alternative, this luciferase detection method was found much more stable, with a > 3 h half-life, and robust enough for producing dose–response plots with as few as 500 sporozoites/well. A screen of the GHPB resulted in 9 hits with selective activity against *P. berghei* liver schizonts, including MMV674132 which exhibited 30.2 nM potency. Retrospective analyses show excellent predictive value for both anti-malarial activity and cytotoxicity.

**Conclusions:**

This method is suitable for high-throughput screening at a cost nearly 20-fold less than using commercial luciferase detection kits, thereby enabling larger liver stage anti-malarial screens and hit optimization make-test cycles. Further optimization of the hits detected using this protocol is ongoing.

**Supplementary Information:**

The online version contains supplementary material available at 10.1186/s12936-024-05155-y.

## Background

Malaria continues to afflict half of the world’s population, predominantly in tropical regions such as Africa, South America, and Southeast Asia [1]. The disease initially causes low-grade fever, chills, and muscle aches. However, as the disease progresses, symptoms worsen to include high fever and exhaustion, leading to severe malaria, which includes anaemia, damage of the organs, and possibly death [2]. Caused by the parasite of the genus *Plasmodium*, *Plasmodium falciparum* and *Plasmodium vivax* are the most widespread, with *P. falciparum* accounting for 99.7% of malaria cases in Africa and *P. vivax* accounting for 46% cases in South America and Southeast Asia [1].

*Anopheles* mosquitoes are the vectors of *Plasmodium* [2]. During a mosquito bite, the mosquito draws host blood while also injecting its saliva. If the mosquito is carrying *Plasmodium*, the highly motile and infective form of the parasite, termed ‘sporozoites’, are also injected into the skin. These sporozoites then travel to the liver through the lymphatic system to infect hepatocytes. Once a hepatocyte is infected, the sporozoite replicates through asexual fission, resulting in thousands of parasites, termed ‘merozoites’, that will burst out of the hepatocyte and enter the bloodstream to infect red blood cells [3]. However, for *P. vivax* and *Plasmodium ovale,* some sporozoites can become a dormant form of the parasite, termed ‘hypnozoites’, that dwell in the liver for months or years before reactivating to cause a relapse blood infection [4].

The two most effective measures against malaria have been the usage of insecticide and the administration of drugs, however, the rapid rise of resistance to both raises concerns about their long-term effectiveness [5]. To continue malaria control and elimination, target candidate profiles (TCPs), which are requirements for specific types of new anti-malarials, are used to guide new anti-malarial development [6]. While much of anti-malarial discovery today is focused on the symptom-causing blood stage (TCP1), the liver stage is regarded as an important therapeutic target for *P. vivax* hypnozoites (TCP3) and a chemopreventive target for *P. falciparum* and *P. vivax* (TCP4). Because of the cost and burdensome logistics of *P. falciparum* and *P. vivax* liver stage assays, liver stage-active compounds are typically first characterized using the well-established *Plasmodium berghei* murine malaria liver model in which HepG2 human hepatoma cells are infected with *P. berghei* sporozoites, treated with test compounds, and then assessed for activity at 44-48 h post-infection [7, 8].

Immunofluorescence is one method of measuring parasite inhibition in liver stage assays. Following this method, cells are fixed, parasites are stained with antibodies, a DNA stain is added to label both the hepatic and parasite nuclei, and high content imaging (HCI) is used to quantify parasite growth [9]. While this method is effective, it also consumes much time and invaluable resources, such as the detection antibodies. Another method of measuring *P. berghei* liver schizont formation is through the usage of luciferase assays. *Photinus pyralis* fireflies contain the *luciferase* gene. This gene codes for the luciferase enzyme which binds to the substrate d-luciferin, causing d-luciferin to undergo oxidative decarboxylation to form oxyluciferin and simultaneously emit energy in the form of light photons. Luciferase has been transgenically engineered into numerous cell-based systems as a reporter for gene expression, cell viability, in vivo imaging, and other applications [10]. One strain of *P. berghei*, PbGFP-Luc_con_ [11], has been used for over a decade to screen thousands of microtitre plates of test compounds, resulting in several new classes of liver stage active anti-malarials. However, a hindrance to luciferase assays is the cost of commercially-sourced detection reagents.

A recent report describes the use of common laboratory reagents and d-luciferin to generate an in-house firefly luciferase assay reagent (FLAR) [12]. In this work, this protocol is incorporated into a *P. berghei* screening platform and then used for screening the Medicines for Malaria Venture (MMV) Global Health Priority Box (GHPB), an open collection of 240 diverse compounds for broad-application drug screening. The optimized method reduces the cost and turnaround of liver stage drug screening and development, thereby potentially expediting the availability of new TCP3 and TCP4 anti-malarials.

## Methods

### HepG2 culture

HepG2 cells (*Homo sapiens* hepatoblastoma, ATCC, cat HB-8065, RRID: CVCL_0027) were cultured in collagen-coated T-75 flasks in media consisting of sugar-free DMEM (Gibco, cat 11966–025) supplemented with 10% FBS (Corning, cat 35–016-CV), 25 mM Glucose (Millipore-Sigma, cat 49163), 1 mM Sodium Pyruvate (Corning, cat 25–000-CI), 1 × penicillin–streptomycin-neomycin mix (Gibco, cat 15640–055), and 2 mM L-glutamine (Gibco, cat 25030–081). Flasks were kept in an incubator at 37 °C and 5% CO_2_.

For seeding microtitre plates, TrpysinLE (Gibco, cat 12605–028) was used to harvest cells from a 60–90% confluent T-75 flask. The cell density was calculated using trypan blue exclusion on a haemocytometer, diluted as needed (see details below), and cells were seeded into collagen-coated 384-well plates (Greiner Bio-One, cat 781956) using a Biomek NXp (Beckman Coulter).

### *Plasmodium* berghei sporozoite production

Luciferase-expressing *P. berghei* ANKA strain GFP-Luc_ama1-eef1a_ (line 1052cl1) were obtained from the Sporocore at UGA as previously described [13]. Sporozoite isolation was performed as previously described using bicarbonate-free RPMI (KD Medical, cat CUS-0645) as the collection buffer [14].

### FLAR stock solutions

The following reagents were obtained and made into the indicated stock solutions in cell culture grade water: 25 mM BD Monolight™ d-luciferin (d-luciferin) potassium salt (BD Biosciences, cat 55677), 25 mM adenosine 5′-triphosphate (ATP) disodium salt hydrate (Millipore-Sigma, cat A26209-1G), 200 mM tricine (VWR, cat 97062–642), 10 mM EDTA (J.T. Baker, cat 8993–01), 50 mM MgSO_4_ (Millipore Sigma, cat M2643), 10 mM MgCO_3_ (VWR, cat 470301–626), and 500 mM DTT (VWR, cat 97,063–760). All stock reagents were stored at 4 °C, except d-luciferin, ATP, and DTT which were aliquoted and stored at − 20 °C. Tricine, MgSO_4_, and MgCO_3_ stocks were all adjusted to have a pH of 7.8. To make a working solution of 1 × FLAR, immediately before the assay endpoint the reagents were mixed to make a solution containing 100 μM d-luciferin, 100 μM EDTA, 125 μM ATP, 1.07 mM MgCO_3_, 2.67 mM MgSO_4_, 20 mM tricine, and 20 mM DTT in cell culture water.

### Sporozoite infection for endpoint optimization studies

Following dissection and quantification of sporozoites, the sporozoite density was set to 250 sporozoites/μL in HepG2 media and serially diluted 1:1 in microcentrifuge tubes using HepG2 media to produce a sporozoite density gradient of 250, 125, 62.5, 31.3, 15.6, 7.81, 3.91, and 1.95 sporozoites/μL. HepG2 cells, seeded at 1.75 × 10^4^ cells/well the day prior, were infected by removing 20 μL of the 40 μL seed volume and adding 20 μL of sporozoite solution to the appropriate rows, resulting in an inoculum of 5.00 × 10^3^, 2.50 × 10^3^, 1.25 × 10^3^, 625, 312, 156, 78, or 39 sporozoites/well (Fig. S1). Two copies of this plate map were seeded and infected, one for the Triton X lysis endpoint and one for the freeze/thaw lysis endpoint. Following infection, plates were spun for 5 min at 200 RCF. After spinning, both plates were stored for 44 h in an incubator at 37 °C and 5% CO_2_.

### Cell lysis and luciferase detection optimization

To test the Triton X cell lysis method, FLAR was prepared at 2 × concentration (40 mM Tricine, 200 µM EDTA, 5.34 mM MgSO4, 2.14 mM MgCO3, 40 mM DTT) and split into 4 aliquots. d-luciferin and ATP were added to aliquots to achieve 2 × (200 µM d-luciferin and 250 µM ATP), 4 × (400 µM d-luciferin and 500 µM ATP), 10 ×  (1 mM d-luciferin and 1.25 mM ATP), and 20 × (2 mM d-luciferin and 2.5 mM ATP) concentration. One of the 384-well plates containing the sporozoite dilution series was dumped of its contents and then 20 µL 0.1% (v/v) Triton X in PBS was added into each well and incubated for 30 min at 37 °C and 5% CO_2_. After Triton X treatment, 20 µL of 2 × FLAR was added into each well of the plate such that 2 × d-luciferin and ATP were added to row A-D (resulting in 1 × final concentration), 4 × to rows E–F (resulting in 2 × final concentration), 10 × to rows I-L (resulting in 5 × final concentration), and 20 × to rows M-P (resulting in 10 × final concentration). To test the freeze/thaw lysis method 1 × FLAR was prepared and split into 4 aliquots. d-luciferin and ATP were added to aliquots to achieve 1 ×  (100 µM d-luciferin and 125 µM ATP), 2  × (200 µM d-luciferin and 250 µM ATP), 5  × (500 µM d-luciferin and 625 µM ATP), and 10  × (1 mM d-luciferin and 1.25 mM ATP) concentrations. To test the freeze/thaw method, the second 384 well plate containing the sporozoite dilution series was dumped of its contents. It was then placed in a − 80 °C freezer for 15 min and then thawed in an incubator at 37 °C for 15 min. After thawing, 40 µL of 2 × FLAR was added into each well of the plate such that 1 ×  d-luciferin and ATP were added to row A-D, 2 × to rows E–F, 50 × to rows I-L, and 10 × to rows M-P. The plates were then placed in a SpectraMax i3x plate reader (Molecular Devices), and luminescence quantified for 500 ms.

### Infection, treatment, and endpoints for legacy anti-malarial in dose–response plates

HepG2 seeding (1.75 × 10^4^ cells/well), salivary gland dissection, sporozoite quantification, and sporozoite dilution were performed as described above. To test the titration of sporozoite in dose–response format with MMV390048, 20 µL of a 25 sporozoite/μL solution in HepG2 media was added to columns 1–8 of 384-well plates (inoculum of 500 sporozoites/well), 20 μL of 50 sporozoites/μL was added into columns 9–16 (inoculum of 1000 sporozoites/well), and 20 μL of 100 sporozoites/μL was added into columns 17–24 (inoculum of 2000 sporozoites/well). To test legacy anti-malarials, two independent experiments were performed for each endpoint (FLAR or HCI), each with an independent production run of *P. berghei* sporozoites. These runs were performed with the maximum number of sporozoites available, which resulted in an inoculum of 1.28 × 10^3^ and 1.96 × 10^3^ sporozoites/well for the FLAR replicates and 1.66 × 10^3^ and 2.00 × 10^3^ sporozoites/well for HCI replicates. Infected plates were spun for 5 min at 200 RCF and kept for 3 h at 37 °C and 5% CO_2_ prior to compound treatment.

Compound treatment was performed as previously described [14]. Dose–response source plates containing 5 μL of 1000 × test compounds in a serial dilution in DMSO were prepared in low volume 384-well plates using a Biomek 4000 (Beckman Coulter). Assay plates were treated by transferring 40 nL from screening or dose–response source plates using a pin tool (V&P Scientific) affixed to a Biomek NXp, resulting in a final test concentration of 1 × in media.

After 44 h, dose–response plates designated for the FLAR endpoint underwent the freeze/thaw lysis method before detection with 1 × FLAR completed with 1 ×  d-luciferin and ATP endpoint described above. Plates designated for the HCI endpoint were fixed with 4% paraformaldehyde (Thermo Scientific, cat 043368.9 M) in PBS for 20 min. Following fixation, the plate was washed twice by adding and then dumping 20 µL of PBS/well. Plates were then stained with 50 ng/mL of mouse anti-*Plasmodium* glyceraldehyde 3-phosphate dehydrogenase (GAPDH) (European Malaria Reagent Repository, cat 13.3) diluted in a 0.3% Triton X and 1% BSA permeabilization and blocking stain buffer overnight at 4 °C. Following three washes with PBS, plates were stained with 2 µg/mL of goat anti-mouse AlexaFluor 488 (Invitrogen, cat A11001) diluted in stain buffer and again incubated overnight at 4 °C. Following three washes with PBS, plates were counterstained with 10 µg/mL Hoechst 33342 (Invitrogen, cat H21492) for 30 min before two washes. Plates were imaged on an ImageXpress Micro Confocal high content system (Molecular Devices). Raw values (either parasite growth area for HCI or Relative Light Units (RLU) for FLAR) were normalized to the positive (MMV390048) or negative (DMSO) controls using the equation:

$$\% Inhibition\; = \,100 \times \left( {\frac{{Raw\;Data{-}Average\;Negative\;Control}}{{Average\;Positive\;Control{-}Average\;Negative\;Control}}} \right)$$

and curve fitting was performed with CDD Vault using the Levenberg–Marquardt algorithm [15, 16]. Potency data from all plates were collected, visualized, and analysed using GraphPad Prism (Version 10.0.3). Potency values for legacy anti-malarials obtained from our FLAR endpoint were compared to those previously reported from a 1536-well plate format using a Bright-Glo™ luciferase detection protocol [17].

### HepG2 seed density optimization, comparison to Bright-Glo™, and signal stability

The ideal HepG2 seed density for optimal luciferase signal was determined by seeding a 384-well plate with a gradient of cells. Following trypsin treatment, the HepG2 cell density was adjusted to 400 cells/µL and a 16-channel pipettor was used to add 5 µL of cells to columns 1 and 2, 7.5 µL to columns 3 and 4, 10 µL to columns 5 and 6, 12.5 µL to columns 7 and 8, 15 µL to columns 9 and 10, 17.5 µL to columns 11 and 12, 20 µL to columns 13 and 14, 22.5 µL to columns 15 and 16, 25 µL to columns 17 and 18, 31.25 µL to columns 19 and 20, 37.5 µL to columns 21 and 22, and 43.75 µL to columns 23 and 24, thereby delivering 2 × 10^3^, 3 × 10^3^, 4 × 10^3^, 5 × 10^3^, 6 × 10^3^, 7 × 10^3^, 8 × 10^3^, 9 × 10^3^, 1 × 10^4^, 1.25 × 10^4^, 1.5 × 10^4^, or 1.75 × 10^4^ cells/well, respectively. Additional media was added to each column to make the total volume/well 40 µL. The next day, sporozoites were harvested as above and the maximum number of sporozoites were infected into wells, resulting in an inoculum of 1.224 × 10^3^ sporozoites/well for replicate 1 and 1.333 × 10^3^ sporozoite/well for replicate 2. To compare the dynamic range and variability with the FLAR and Bright-Glo™ endpoints, wells designated for either endpoint were then treated with 0.1% v/v DMSO in media and additional set of wells designated for the Bright-Glo™ endpoint were treated with 1 µM MMV390048 in media. After 44 h, media was dumped from the plate and cells lysed using the freeze/thaw method as above. Luminescence signal from four rows of the plate were detected using 40 µL 1 × FLAR as above, and four other rows of the same plate were detected using 20 µL Bright-Glo™ (Promega, cat E2610) prepared by adding 10 mL of the provided buffer to 1 vial of the provided substrate at room temperature, per manufacturer’s instructions. Plates were read immediately as above to ascertain the effect of seed density on signal and variance by endpoint, and then read every 3 min for 3 h in a kinetic experiment to characterize signal stability.

### GHPB screen and dose–response confirmation

The GHPB is an open-access collection of compounds (80 for vector control, 80 for zoonotic and neglected diseases, 80 for drug resistant malaria) obtained from MMV. The library was supplied in a 96-well microtitre plate and moved into a low-volume 384-well plate (Greiner Bio-One, cat 784,261) using a Biomek NXp to transfer 5 μL of 1 mM compound into the destination wells. MMV390048 (1 µM) was used for the positive control wells and DMSO (0.1% v/v) was used for the negative control wells. The GHPB was screened by seeding 5 × 10^3^ cells/well which, after 24 h, were infected with a maximum available inoculum of 1.44 × 10^3^ sporozoites/well. Three hours post-infection, plates were treated with a pin tool as described above. After 44 h, media was dumped from the plate, lysed using the freeze/thaw method, and luciferase signal was detected with 1 × FLAR as described above.

To assess the general cytotoxicity of GHPB compounds, HepG2 cells were cultured in media containing galactose instead of glucose to avoid false negatives due to the Crabtree effect [18]. Following propagation of HepG2 in T-75 flasks with glucose-containing media as above, media was aspirated, the cell monolayer was washed with PBS, and then cells were trypsinized as above. Released cells were then resuspended in media containing glucose-free DMEM (Gibco, cat 11,966–025) supplemented with 10% FBS (Corning, cat 35–016-CV), 10 mM galactose (Sigma, cat G5388), 1 mM sodium pyruvate (Corning, cat 25–000-CI), 1 × penicillin–streptomycin-neomycin mix (Gibco, cat 15,640–055), and 2 mM L-glutamine (Gibco, cat 25,030–081). The cell density was calculated using trypan blue exclusion on a haemocytometer, diluted to 50 cells/µL, and 40 µL of cells were seeded into collagen-coated 384-well plates (Greiner Bio-One, cat 781,956) using a Biomek NXp (Beckman Coulter), resulting in 2 × 10^3^ cells/well. The day after seeding, plates were treated with a pin tool as above and cultured for 72 h. Plates were then fixed with 4% paraformaldehyde, stained with 10 µg/mL Hoechst 33,342, and imaged on an ImageXpress Micro Confocal. Hepatic nuclei were quantified using MetaXpress (Molecular Devices) image analysis software. Both *P. berghei* liver schizont and HepG2 cytotoxicity data were loaded into CDD Vault for normalization and hit selection as described above.

A total of 35 compounds were identified for resupply of fresh powder for confirmation in dose–response assays. This included 6 hit compounds with > 80% inhibition of *P. berghei* and < 15% inhibition of HepG2 cells. The other 29 compounds were inactive in the primary screen but selected to characterize the *P. berghei* liver schizont and HepG2 cytotoxicity assays’ positive and negative predictive values. Powders were diluted to 50 mM in DMSO and plated in 1000 × source plates as described above. MMV390048 and puromycin were plated as the positive control for *P. berghei* liver schizont activity and cytotoxicity, respectively. Two independent experiments using 1 × FLAR and 5 × 10^3^ cells/well were performed to assess the potency of these compounds against *P. berghei* liver schizonts, each with an independent production run of *P. berghei* sporozoites. These runs were performed with the maximum number of sporozoites available, which resulted in an inoculum of 1.22 × 10^3^ and 1.33 × 10^3^ sporozoites/well. Separately, two independent runs of the HepG2 cultured in galactose, assayed as described above, were used to assess the cytotoxicity potency and selectivity indices for these compounds. Both *P. berghei* liver schizont and HepG2 cytotoxicity data were loaded into CDD Vault for normalization, curve fitting, and EC_50_ calculations as described above. Receiver-operator characteristic (ROC) curves were generated using Graphpad Prism and area under the curve (AUC) was calculated using the Wilson/Brown method for both *P. berghei* liver schizont activity and HepG2 cytotoxicity [19]. For *P. berghei* ROC classification, hits found active in dose–response with an EC_50_ < 0.333 µM were considered as positives and compounds with an EC_50_ > 0.333 µM were considered negative as they would be expected to be only partially active or inactive in a 1 µM primary screen (ie see MMV689635 below). For cytotoxicity ROC classification, hits found cytotoxic in dose–response with a CC_50_ < 2 µM were considered toxic as even a small loss of hepatic nuclei (ie, 15% or more) is indicative of host cell inhibition and thus would be detected as partially active in the 1 µM primary screen.

## Results

**Fig. 1:**
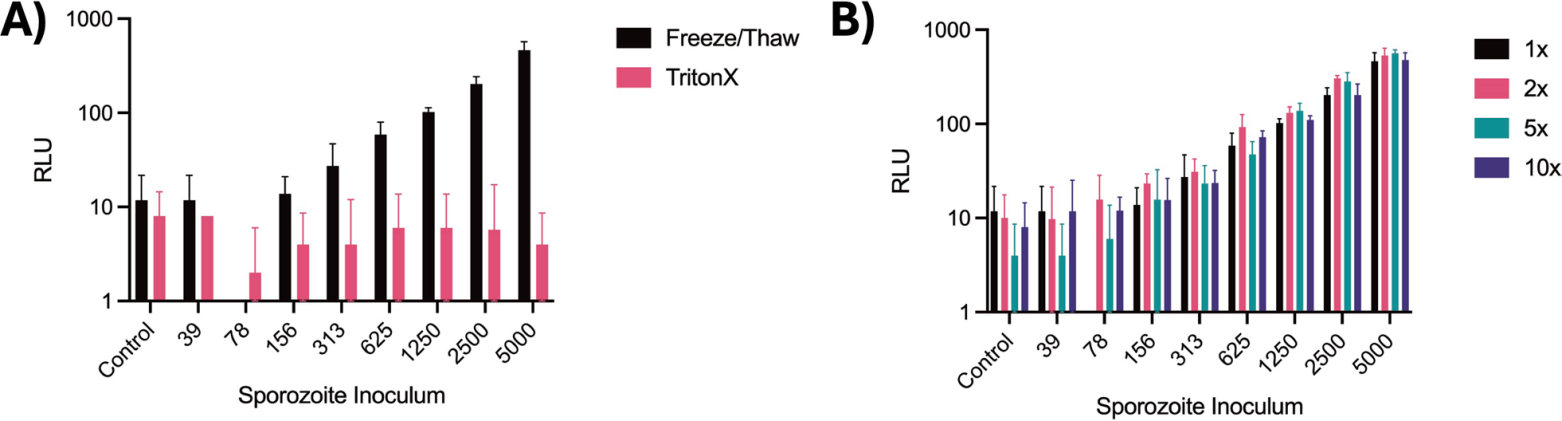
Optimization of conditions for a luciferase endpoint with in-house reagents (FLAR). **A** Impact of lysis method on relative luminescence unit (RLU) signal at 30 min post addition of 1 × FLAR. **B** Impact of sporozoite inoculum and different concentrations of ATP and d-luciferin in FLAR buffer on RLU signal at 30 min post FLAR addition. **A**, **B** Bars represent S.D. of four replicate wells per condition. Data shown are from one independent experiment representative of two independent experiments

The previously reported FLAR protocol was adapted for detecting *P. berghei* liver stage schizont growth by first simultaneously testing a range of FLAR concentrations, two different methods for cell lysis, and a range of sporozoite inocula to generate a gradient of schizonts within wells of a 384-well plate (Fig. S1) [12]. To test the effectiveness of each lysis method, two replicate plates were started and used for either lysis via freeze/thaw or Triton X. Much higher RLUs were recorded using the freeze/thaw lysis method (Fig. 1A) and robust signal over background became apparent with an inoculum as low as 625 sporozoites/well (Fig. 1B). The concentration of FLAR did not appear to affect overall signal or well-well variability (Fig. 1B). From these results, the freeze/thaw lysis and 1 × FLAR were used for subsequent experiments (Table 1).

**Table 1:** Preparation of FLAR buffer

Reagent	[Stock]	[Working]	For 20ml FLAR
Tricine	200 mM	20 mM	2 ml
MgSO4	50 mM	2.67 mM	1.068 ml
MgCO3	10 mM	1.07 mM	2.14 ml
EDTA	10 mM	100 µM	200 µl
DTT	500 mM	20 mM	*800 µl*
d-luciferin	25 mM	100 µM	*80 µl*
ATP	25 mM	125 µM	100 µl
Cell Culture Water			13.6 ml

Buffer is made immediately before the assay endpoint. A 20 mL volume is sufficient for addition of up to 200 µL per well of a 96-well plate, 50 µL per well of a 384-well plate, or 20 µL per well of a 1536-well plate (with a margin remaining)

**Fig. 2:**
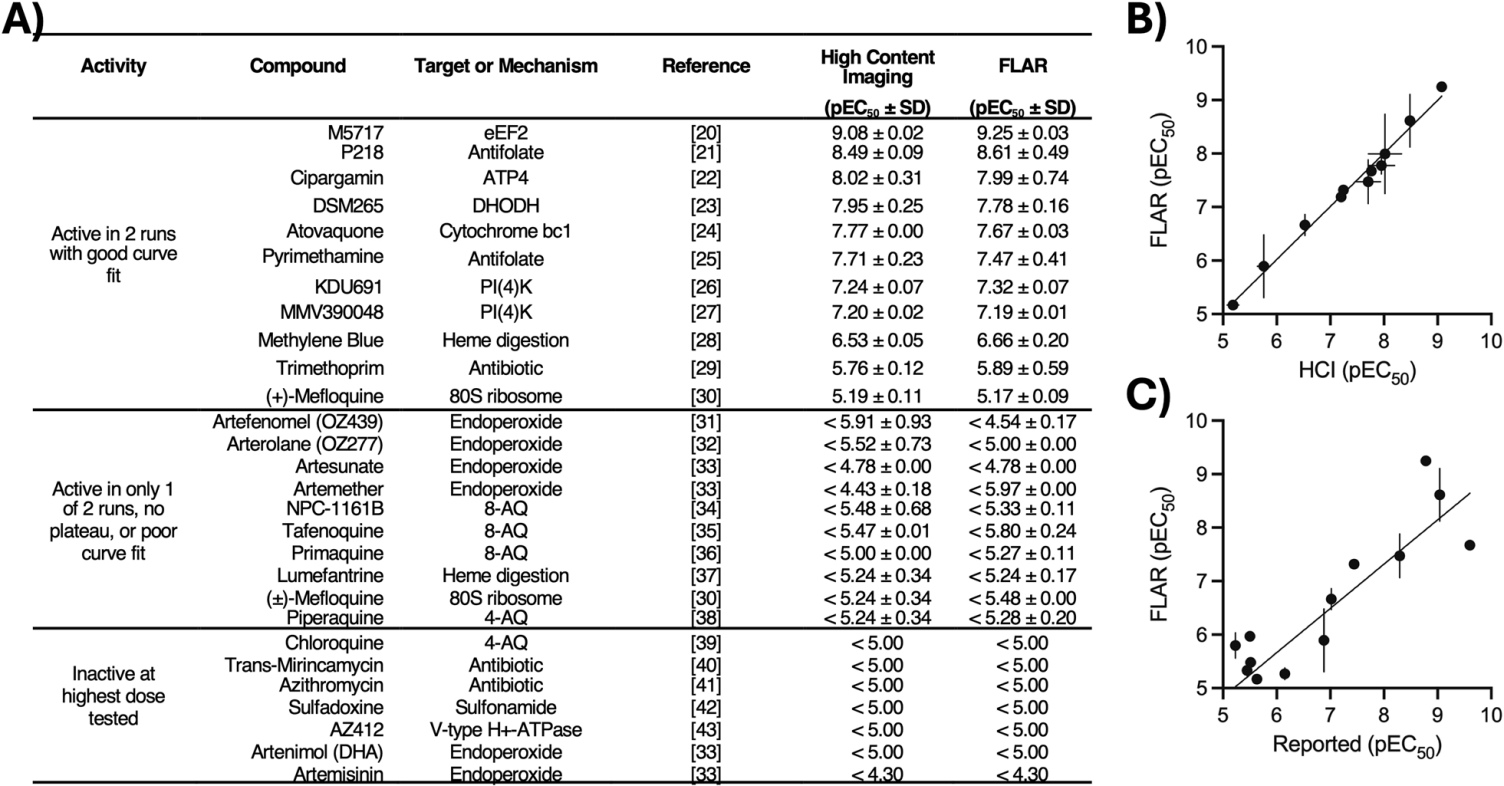
Comparison of high content imaging (HCI) and FLAR endpoints for generating potency data. **A** Table of pEC_50_ values for legacy and developmental anti-malarials tested. pEC50 is the negative log of potency in M (ie a pEC_50_ of 6 = an EC_50_ of 1 µM and a pEC_50_ of 9 = an EC_50_ of 1 nM). Values are the average and S.D. from two independent experiments (runs). Anti-malarial potencies are grouped by those active in both runs, those active in only one run or producing poor curve fits, and those inactive at the highest dose tested in both runs. eEF2, elongation Factor 2 inhibitor; DHODH, dihydroorotate dehydrogenase inhibitor, PI(4)K, phosphatidylinositol-4-OH kinase inhibitor; 8-AQ, 8-aminoquinoline, 4-AQ, 4-aminoquinoline; DHA, dihydroartemisinin. The “Target or Mechanism” column provides a reference for the possible or demonstrated mode of action and is not meant to be exhaustive or conclusive. **B** Plot of potency values obtained from the HCI versus FLAR endpoints. Line represents a simple linear regression (Y = 0.9919*X + 0.06728, R^2^ = 0.9863). **C** Plot of potency values obtained from FLAR endpoint and those reported using a luciferase protocol in a 1536-well plate format [17]. Line represents a simple linear regression (Y = 0.8275*X + 0.6979, R^2^ = 0.7546). **B**, **C** Points and bars represent the average and S.D. of the pEC_50_’s calculated from two independent experiments for the HCI and FLAR endpoints

To demonstrate the effectiveness of using FLAR as an alternative to HCI, legacy anti-malarials were tested in full dose-response assays using both detection methods. To begin, the FLAR protocol was used to measure the potency of the multi-stage active phosphatidylinositol-4-kinase (PI4K) inhibitor MMV390048 in assays started with an inoculum of 0.5 × 10^3^, 1.0 × 10^3^, or 2.0 × 10^3^ sporozoites. Excellent dose–response curves and similar EC_50_ calculations were noted from assays started with all three inocula, indicating this range was suitable for additional studies (Fig. S2A). Next, a set of 28 legacy, clinically used, or in-development anti-malarials were selected and assayed with the maximum inoculum based on the number of sporozoites harvested from salivary gland dissections (which are variable and somewhat unpredictable across production runs). After testing these 28 inhibitors in two independent experiments using the FLAR endpoint and two independent experiments using the HCI endpoint, the two endpoints resulted in nearly identical potency calculations (Fig. 2, simple linear regression, Y = 0.9919*X + 0.06728, R^2^ = 0.9863). Of note, the MMV390048 control was one of the 28 in-development anti-malarials tested and produced a robust dose–response curve using an inoculum of 1.27 × 10^3^ (run 1) or 1.96 × 10^3^ (run 2) sporozoites/well (Fig. S2B).

The activity of the 28 validation compounds can be summarized into three groups (Fig. 2A) [20–43]. First were those resulting in a full dose–response curve in both independent experiments. This group included the in-development inhibitors M5717, P218, cipargamin, and DSM265, which were found as the most potent of the entire set. Like MMV390048, the PI4K inhibitor KDU691 killed liver stage schizonts at high nanomolar potency. Also included were legacy anti-malarials pyrimethamine, atovaquone, methylene blue, and enantiopure mefloquine. The second group were those which were either potent at the highest dose only, which resulted in a poor curve fit, or were active in only one of two independent experiments. Interestingly, this group contained the endoperoxides OZ439, OZ277, artesunate, and artemether which are not known for liver stage activity but are also cytotoxic at higher doses. The 8-aminoquinolines (8-AQs) also showed weak activity in this group. While 8-AQs are primarily used for the radical cure of *P. vivax* malaria, which includes liver-resident hypnozoites, these compounds often show poor activity in vitro as they must be activated by hepatic cytochrome P450 enzymes which are not highly expressed in hepatoma cells [36, 44]. Several other legacy anti-malarials were found inactive in both runs and placed in the third group. Altogether, these results were highly congruent with a previous report on the *P. berghei* liver stage activity of these drugs using a commercial luciferase reagent in a 1536-well plate format (Fig. 2C) [17].

**Fig. 3:**
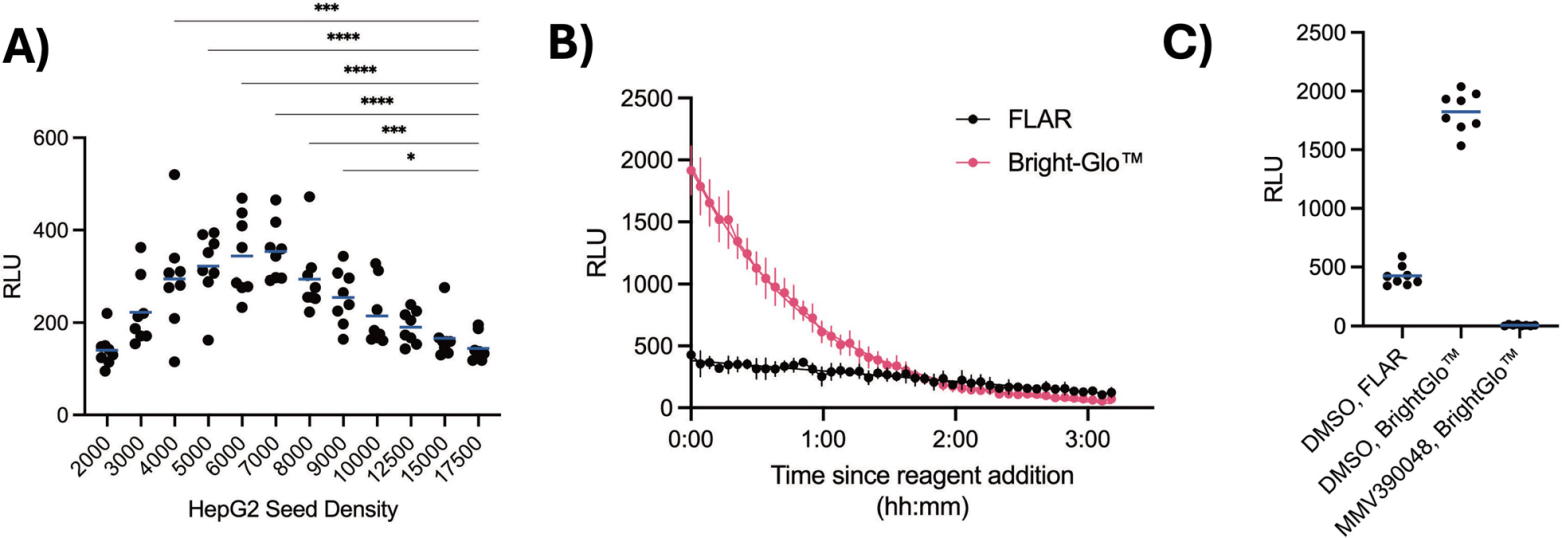
Optimization of HepG2 seeding conditions for robust *P. berghei* luciferase signal. **A** Impact of seed density on RLU signal. Individual datapoints represent 8 replicate wells read immediately after FLAR addition. Blue lines represent mean. Significance determined by one-way ANOVA, F(11,84) = 10.01, p < 0.0001, with Dunnett’s multiple comparisons to the 17,500 condition, *p < 0.05, ***p < 0.0005, ****p < 0.0001, and nonsignificant comparisons have no indication. **B** Kinetic read of *P. berghei* luciferase signal in wells seeded with 5 × 10^3^ HepG2 cells/well and detected with either 1 × FLAR or Bright-Glo™. Bars represent S.D. of 8 replicate wells. Curves were fitted using one phase exponential decay (R^2^ = 0.9390 for FLAR and 0.9975 for BrightGlo™), half-life was > 3 h for FLAR and 38 min for BrightGlo™. **C** *P. berghei* luciferase signal comparison of the first timepoint of (**B**) using 1 × FLAR or Bright-Glo™. Wells were treated with 0.1% v/v DMSO or 1 µM MMV390048 as indicated. Blue lines represent mean. **A**–**C** Data shown are from one independent experiment representative of two independent experiments

Before proceeding with using FLAR for screening compound libraries, in which each well contains a different test compound tested at a single concentration, additional optimizations were considered to boost signal as the RLUs being generated in dose–response assays were lower than those obtained in previous studies [45]. The HepG2 seed density was prioritized for optimization as the density routinely used in previous reports, 1.75 × 10^4^ cells/well in a 384-well microtitre plate format, which is equivalent to 1.61 × 10^3^ cells/mm^2^ well bottom area, resulted in a complete monolayer immediately after seeding [46]. Given the assay requires one day before infection and two days before the endpoint, this seed density likely leads to infected cells being lost to overcrowding, thereby decreasing the RLU signal. A titration of the number of HepG2 seeded per well revealed seed density affects signal, with a far lower seed density of 5 × 10^3^ cells/well (equivalent to 459 cells/mm^2^ well bottom area) producing higher RLUs compared to 1.75 × 10^4^ cells/well (one-way ANOVA, F(11,84) = 10.01, p < 0.0001, with Dunnett’s multiple comparisons, p < 0.0001) (Fig. 3A). With a fully optimized protocol, signal stability, the dynamic range, and variance of 1 × FLAR were characterized and compared to Bright-Glo™ luciferase detection reagent, which is commonly used in *P. berghei* liver stage screening assays [17]. While Bright-Glo™ generated higher RLUs than FLAR immediately after reagent addition, using a kinetic read over 3 h, Bright-Glo™ signal decreased rapidly (t_1/2_ = 38 min) compared to FLAR (t_1/2_ > 3 h) (Fig. 3B). Further analysis of the reading immediately following reagent addition revealed Bright-Glo™ generated 4.3-fold more signal than FLAR but also higher variance (x̄ = 1823, σ = 170.6, S/N = 10.7 for Bright-Glo™ and x̄ = 425.3, σ = 85.26, S/N = 4.99 for FLAR).

**Fig. 4:**
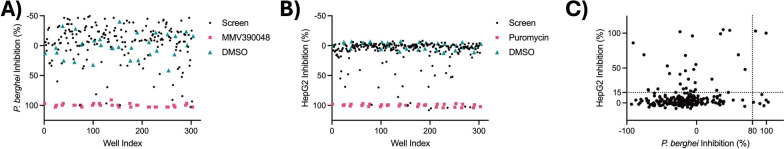
GHPB single point screen. Compounds were tested at 1 µM against (**A**) the *P. berghei* liver schizonts and (**B**) HepG2 cells culture in galactose media. **C** For confirmation, 35 compounds, including 6 hits with > 80% inhibition of *P. berghei* liver schizonts and < 15% inhibition of HepG2 cells (lower right quadrant), were resupplied for dose–response assays

The fully optimized FLAR assay was next used to test the GHPB, an open-source collection of compounds targeting parasite vectors, zoonotic diseases, and drug-resistant *P. falciparum,* at 1 µM [47]. The GHPB was counter-screened for general cytotoxicity using HepG2 cells cultured in galactose media. This version of cytotoxicity assay is important for detecting mitochondrial inhibitors, as cells cultured in glucose can dispense with mitochondrial respiration and survive on glycolysis alone [18]. The screens detected 8 compounds with > 80% inhibition of *P. berghei* liver schizonts, which is a relatively high hit rate of 3.3%, but also 38 compounds with > 15% cytotoxicity, which included 2 of the 8 *P. berghei* liver schizont hits (Fig. 4). To characterize the predictive value of these assays, which requires testing of hit and inactive compounds in dose–response assays, 35 compounds were selected to be resupplied from powder. This set including the 6 hits that appeared active (> 80% inhibition against *P. berghei* liver schizonts) and selective (< 15% inhibition of HepG2) in the primary screens, as well as 29 that were inactive and non-cytotoxic at 1 µM.

**Fig. 5:**
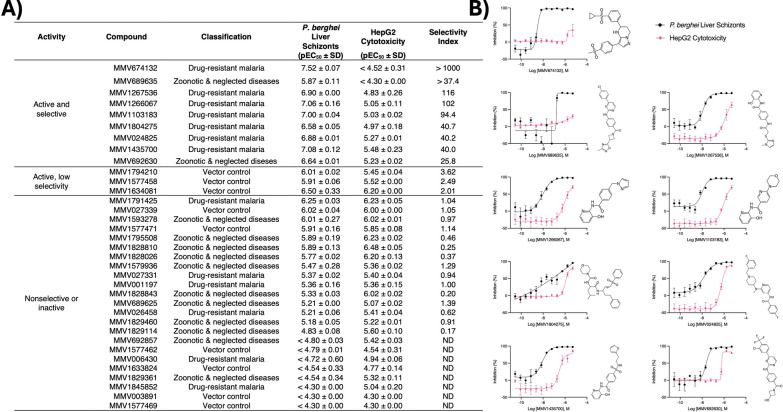
Results table, potencies, and structures of selective GHPB hits. **A** Table of pEC_50_ values for GHPB compounds. pEC50 is the negative log of potency in M (ie a pEC_50_ of 6 = an EC_50_ of 1 µM and a pEC_50_ of 9 = an EC_50_ of 1 nM). Values are the average and S.D. from two independent experiments (runs). Anti-malarial potencies are grouped as selectively active, active but with low selectivity, and nonselective or inactive. **B** Dose–response charts and structures for active and selective hits. Bars represent S.E.M. of two replicate wells at each dose from each of two independent experiments charted together

The set of 35 hit and inactive compounds were tested against *P. berghei* liver schizonts and HepG2 cells in dose–response from 50 µM—a much higher dose than the primary screen dose of 1 µM—to ensure calculation of potency at doses just higher than the primary screen dose and, therefore, a better understanding of selectivity. All 6 of the resupplied hits that were active and selective in the primary screen (MMV1103183, MMV024825, MMV674132, MMV692630, MMV1266067, and MMV1435700) confirmed to be active and selective in confirmation runs (Fig. 5). An additional three resupplied compounds which were not considered hits in the primary screen (MMV1267536, MMV1804275, and MMV689635) were also active and selective in confirmation runs. Upon further analysis, the EC_50_ of MMV689635 against *P. berghei* liver schizonts was just above 1 µM, explaining why it was not also classified as hits from the primary screen. MMV1804275 and MMV1267536, which yielded 66% and 69.1% inhibition of *P. berghei* liver schizonts in the primary screen, respectively, demonstrate the hit threshold of 80% could be lowered to ensure similar true positives with partial activity in the primary screen are not missed. The remaining 26 resupplied compounds were found either toxic or inactive in dose–response assays. Of the confirmed hits, MMV674132, an imidazopyridazine previously described as having 44 nM potency against *P. falciparum* asexual blood stages and 1–3 µM potency against *P. falciparum* gametocytes, showed the most potency against *P. berghei* liver schizonts (EC_50_ = 30.2 nM) and only marginal cytotoxicity at doses above 10 µM, resulting in a selectivity index of > 1000 (Fig. 5B) [48]. Of note, the MMV390048 control resulted in similar potency throughout assay optimization and validation (Fig. S2C).

**Fig. 6:**
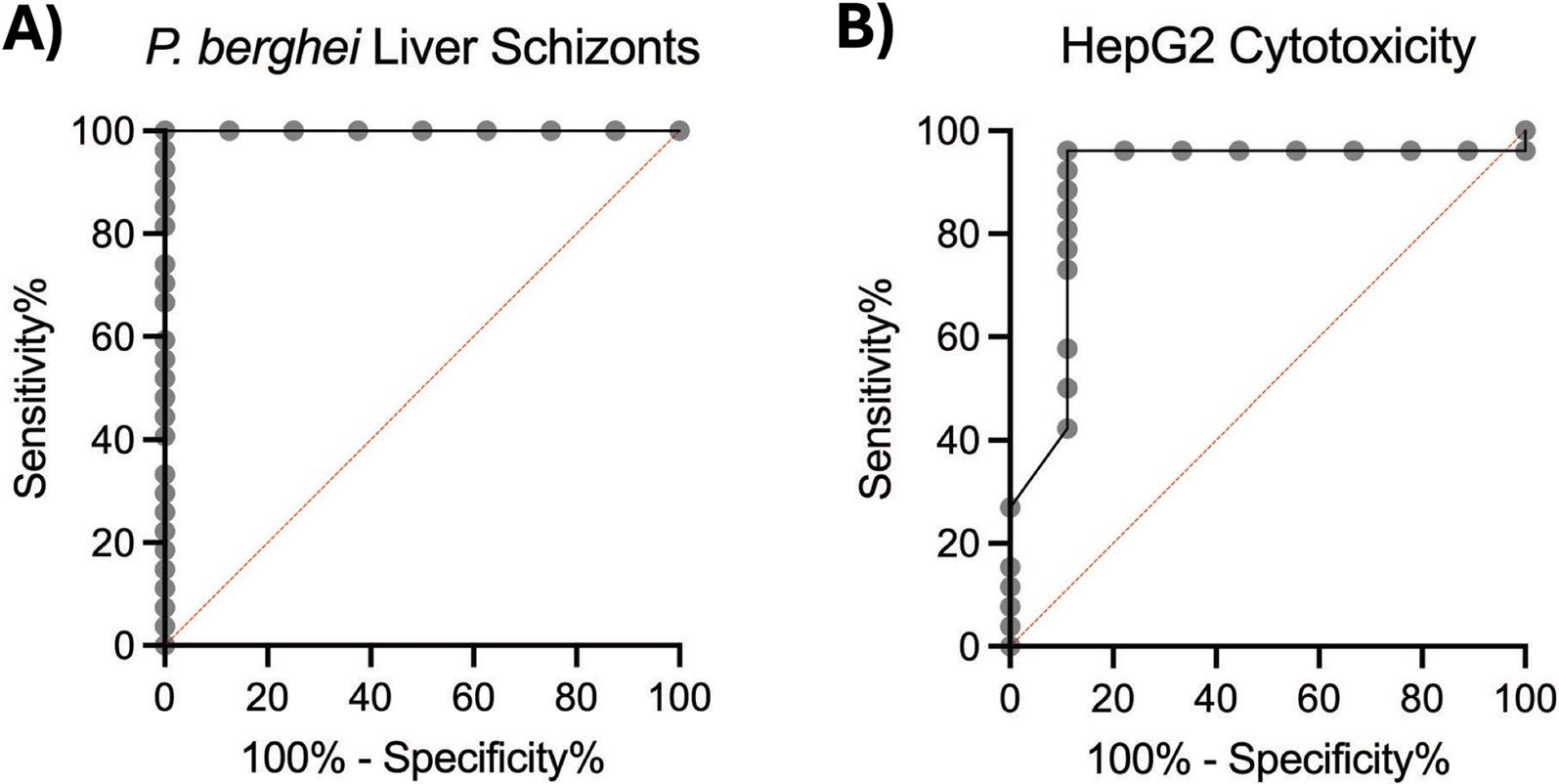
Receiver-operator characteristic (ROC) curves of GHPB compounds tested in *P. berghei* and cytotoxicity assays. ROC curves indicate the sensitivity and specificity of an assay based on a range of inhibition values from the primary screen (grey circles). The red line indicates a random assay. **A** ROC curve for *P. berghei* using FLAR, AUC = 1.0 (95% CI 1.0–1.0). B) ROC curve for HepG2 cytotoxicity using galactose media, AUC = 0.8932 (95% CI 0.7465–1.000). AUCs were calculated using the Wilson/Brown method

Lastly, retrospective analyses were used to show how the primary *P. berghei* and cytotoxicity screens performed. Using an inoculum of 1.44 × 10^3^ sporozoites/well, RLUs ranged from 159-370 (x̄ = 266, σ = 51.6) in the DMSO control wells and 0–31 (x̄ = 7.22, σ = 7.32) in the MMV390048 positive control wells. For high-throughput screening purposes, this led to a good coefficient of variance (CV = 19.4), Z’-factor (Z’ = 0.316), and dynamic range (S/N = 36.8). A ROC analysis confirmed the *P. berghei* assay was capable of detecting hits with an EC_50_ of < 0.333 µM (AUC of 1.0 (95% CI 1.0–1.0, p < 0.0001) (Fig. 6A). The HepG2 cytotoxicity primary screen was also predictive, with an excellent robust Z-factor (Z’ = 0.733) and ROC AUC of 0.8932 (95% CI 0.7465–1.000, p = 0.0005) (Fig. 6B). Taken together, these results show this screening workflow can be used for high-throughput screening against *P. berghei* liver schizonts with only 1.44 × 10^3^ sporozoites/well, albeit using more sporozoites will increase the signal and generate higher Z-factors if desired.

## Discussion

Over the past 20 years, several reports describe large anti-malarial screening and hit compound development efforts using *P. berghei* liver stage parasites to characterize liver stage activity [49–56]. Resultingly, novel classes of inhibitors with liver stage activity, including M5717, cipargamin, DSM265, and MMV390048, are now in late-stage development or clinical trials [20, 22, 23, 27]. However, given the history of resistance to new anti-malarials, the anti-malarial development pipeline should continue supporting the discovery and development of new classes against novel targets [6]. While millions of compounds have been tested in *P. falciparum* blood stage assays, the largest liver stage screen to date was 5 × 10^5^ compounds against luciferase-expressing *P. berghei* over 18 months using 1603 384-well plates [57]. Following an extensive literature search and review, there appear to be no reports of a *Plasmodium* liver stage screening assay using non-commercial detection reagents. Conversely, at the time of this report, a search in Pubmed yielded 50 other reports describing the implementation of reagents developed by Siebring-van Olst et al. [12].

Optimizations occurred in two phases. At first, HepG2 were seeded at a density of 1.75 × 10^4^ cells/well in a 384-well microtitre plate format, which is equivalent to 1.61 × 10^3^ cells/mm^2^ well bottom area. This number was based on prior optimizations and other *P. berghei* liver stage luciferase assays in a 384-well microtitre plate format [46, 52]. Similar to the approach taken by Siebring-van Olst et al., reagent concentrations and lysis methods were optimized in the first phase [12]. With a working FLAR protocol, the method was then validated using legacy and in-development anti-malarials tested in dose–response, allowing for a direct comparison of the potencies obtained using FLAR to those obtained with HCI and from another luciferase-based assay [17]. While nearly identical potencies were obtained for the legacy anti-malarials using FLAR and HCI, the net RLUs obtained were lower than expected [45]. A second phase of optimization was then used to optimize the HepG2 seed density. Interestingly, a much lower seed density of 5 × 10^3^, which is less than a third the density previously used, led to ideal RLU signal intensities. This simple finding could be impactful for the field as *P. berghei* liver stage assays performed in 96, 384, and 1536-well microtitre plate formats typically also use a much higher density [17, 45, 52].

Optimization studies were concluded by comparing the performance FLAR against a commonly used commercial detection reagent, Bright-Glo™. Bright-Glo™ was found brighter than FLAR but did not provide as stable a signal over time. This is not unexpected as the product use guide for Bright-Glo™ states Bright-Glo™ should be used for signal intensity while another Promega product, Steady-Glo™, is less bright but should be used when a longer half-life is needed [58]. For *P. berghei* liver stage assays, it is possible that Bright-Glo™ is ubiquitously used because only a fraction of hepatocytes are infected with luciferase-expressing parasites, thus signal amplification could be useful. Conversely, a longer half-life could be important when screening larger libraries, where many microtitre plates could be simultaneously subjected to an endpoint using automated liquid handling and then read in sequence over several hours.

Following protocol optimization, FLAR was used to screen the GHPB and confirm hit activity in dose–response assays. A separate HepG2 assay using galactose media was used to also screen the GHPB for cytotoxicity and determine selectivity in dose–response assays. While the Z’ of the primary screen using an inoculum of 1.44 × 10^3^ sporozoites/well was lower (Z’ = 0.316) than Z’ scores previously reported in a 384-well plate format using an inoculum of 4.0 × 10^3^ sporozoites/well and Bright-Glo™ in the endpoint, the assay was robust enough to detect and confirm active compounds [50]. These results show that, while a higher inoculum can be used by individual laboratories to increase the signal, and therefore Z’, of a FLAR-based protocol, a lower inoculum can still result in useful screen data and each laboratory must balance the tradeoff between throughput and signal [50].

Of the 240 compounds in the GHPB, 80 with activity against *P. falciparum* blood stage parasites are considered the “Malaria Box 2” and include 6 dual-active structures with known activity against *P. berghei* liver stage parasites (MMV1262756, MMV1266067, MMV1435700, MMV1103183, MMV1267536, and MMV1167451) [47, 57]. The FLAR primary screen successfully detected 3 of these 6 structures as selective hits. Of the 3 structures not detected, MMV1167451 was reported to have weaker (EC_50_ = 6.3 µM) potency against *P. berghei* liver schizonts and, therefore, was understandably missed when tested at the screening concentration of 1 µM [57]. MMV1267536 was somewhat active in the primary screen (69.1% inhibition at 1 µM) and was therefore selected as a borderline hit for confirmation in dose–response (EC_50_ = 127 nM). MMV1262756 was reported to have sub-micromolar potency (EC_50_ = 105 nM) against *P. berghei* liver schizonts but for an unknown reason was not detected as a hit in the primary screen (− 11.7% inhibition at 1 µM) [57]. Regardless, the screen detected 3 other compounds with previously unreported sub-micromolar activity against *P. berghei*, including one with > 1000-fold selectivity (MMV674132).

Similar to past screens of bioactive libraries, a large proportion of the library was cytotoxic at 1 µM and might be down-prioritized as a starting point for drug development [44]. Even though an HCI endpoint or a viability reagent could have been used to quantify HepG2 viability in the *P. berghei* liver stage screening plates directly, galactose assay-dedicated plates were used for both the primary screen and dose–response confirmation runs. The galactose assay is advantageous because of its ability to detect mitochondrial inhibitors, a lower seed density (2.0 × 10^3^ cells/well) which increases the dynamic range of the assay, an additional 28 h incubation time (72 h for the galactose cytotoxicity assay versus 44 h for the *P. berghei* liver stage assay) useful for detecting slow-acting compounds, and the use of HCI to quantify cytotoxicity phenotypes in otherwise live cells [59]. Of note, unlike using IFA to detect parasites for an HCI-based *P. berghei* liver stage assay, the galactose assay uses only Hoechst 33342 stain as a marker, which is widely available and inexpensive.

Further delving into the workflow and cost of luciferase detection reagents, Bright-Glo™ can be used without first removing media from assay plates. This approach was not tested during optimization studies because previous reports of *P. berghei* liver stage luciferase assays frequently take advantage of a single assay plate to also generate cytotoxicity data by first using a cell viability reporter, reading the plate, dumping the reagents, and then adding luciferase detection reagents [55]. As such, the general workflow for obtaining both the cytotoxicity and efficacy endpoints would be unchanged if using either a commercial product like Bright-Glo™ or FLAR. Because both methods include addition of FLAR to empty plates, only 10 mL of Bright-Glo™ is needed to detect luminescence from a full 96, 384, or 1536-well microtitre plate, while the FLAR protocol was optimized for 20 mL of FLAR to detect luminescence from a similar plate. Despite the higher volume used per well, FLAR was found useful for detecting hits in a single-point screen of the GHPB at reduced cost compared to using a commercial detection reagent. After scaling for the amount of each reagent needed, FLAR reagents cost $8.58 per plate while Bright-Glo™ reagent cost $160 per plate. In the context of the entire assay cost, one of the most expensive components is the *P. berghei-*infected mosquitoes, which can be purchased and shipped from the Sporocore at the University of Georgia for less than $5.00 per mosquito. Accounting for the lower limit of sporozoites harvested per mosquito and sporozoites needed per well, as few as 18 infected mosquitoes are needed per plate, which cost about $87. Thus, the combined cost of sporozoites and detection reagent for one plate when using Bright-Glo™ is $247, and for FLAR $97, of which the detection reagent accounts for 65% and 9% of the net cost, respectively (Table S1). Ideally the FLAR reagent described in this report can be used by other in the anti-malarial drug discovery field and the hits identified from the GHPB can be assessed for further development.

## Supplementary Information

**Supplementary materials 1: Figure S1.** 384 well-plate mapping of optimization assay. “Spz/well” indicates the net number of sporozoites added per well. Concentrations of FLAR were supplemented with 1x, 2x, 5x, or 10 × ATP and D-luciferin. For example, 1 × FLAR had 100 µM D-luciferin and 125 µM ATP, while 2 × FLAR had a two-fold higher concentration of D-luciferin and ATP.

**Supplementary materials 2: Figure S2. **Potency data for the MMV390048 control using the luciferase protocol. A) HepG2 were seeded at 1.75 × 10^4^ cells/well and infected with a titrated inoculum of 500, 1000, or 2000 sporozoites/well to characterize how the potency of the MMV390048 control is affected by inoculum. Data shown are from a single matching experiment including all three inoculums. B) Dose–response plot of MMV390048 control from validation assaysafter seeding HepG2 at 1.75 × 10^4^ cells/well and infecting with a sporozoite inoculum of 1.27 × 10^3^ for run 1 and 1.96 × 10^3^ for run 2. C) Dose–response plot of MMV390048 control from GHPB hit confirmation assaysafter seeding HepG2 at 5.00 × 10^3^ cells/well and infecting with a sporozoite inoculum of 1.22 × 10^3^ for run 1 and 1.33 × 10^3^ for run 2. Bars represent S.E.M. of replicate wells at each dose.

**Supplementary materials 3: Table 1. **Cost analysis per plate for in-house FLAR reagent versus Bright-Glo™.

## Data Availability

The datasets used and/or analysed during the current study are available from the corresponding author on reasonable request. No datasets were generated or analysed during the current study.

## Abbreviations

TCP: Target candidate profile
HCI: High content imaging
FLAR: Firefly luciferase assay reagent
MMV: Medicines for Malaria Venture
GHPB: Global Health Priority Box
GADPH: Glyceraldehyde 3-phosphate dehydrogenase
RLU: Relative luminescence unit
ROC: Receiver-operator characteristic
AUC: Area under curve
PI4K: Phosphatidylinositol-4-kinase
