# Supplementary figures and images for: Screening the Global Health Priority Box against Plasmodium berghei liver stage parasites using an inexpensive luciferase detection protocol
Source: Malar J. 2024 Nov 23;23:357. doi: 10.1186/s12936-024-05155-y (PMC11585928; doi:10.1186/s12936-024-05155-y)

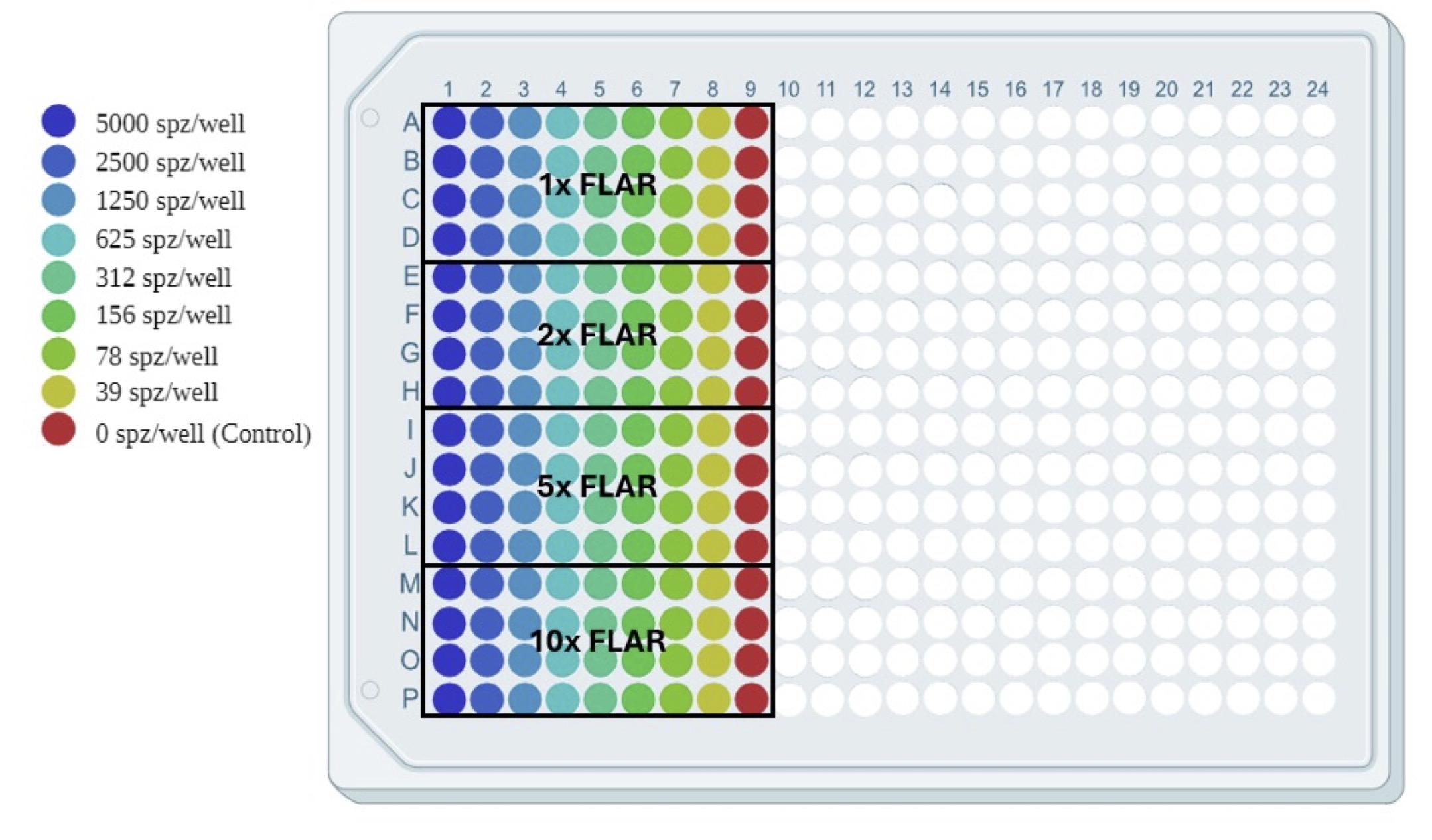

Supplement: Supplementary file 1 — Supplementary materials 1: Figure S1. 384 well-plate mapping of optimization assay. “Spz/well” indicates the net number of sporozoites added per well. Concentrations of FLAR were supplemented with 1x, 2x, 5x, or 10 × ATP and D-luciferin. For example, 1 × FLAR had 100 µM D-luciferin and 125 µM ATP, while 2 × FLAR had a two-fold higher concentration of D-luciferin and ATP. [file 12936_2024_5155_MOESM1_ESM.jpg]

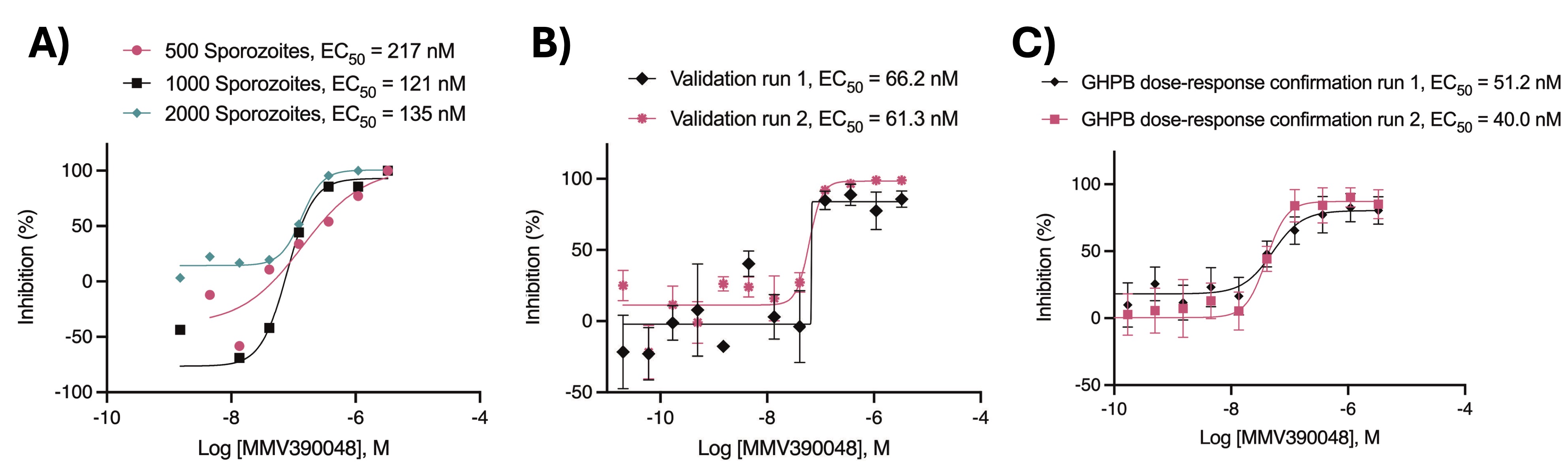

Supplement: Supplementary file 2 — Supplementary materials 2: Figure S2. Potency data for the MMV390048 control using the luciferase protocol. A) HepG2 were seeded at 1.75 × 10^4^ cells/well and infected with a titrated inoculum of 500, 1000, or 2000 sporozoites/well to characterize how the potency of the MMV390048 control is affected by inoculum. Data shown are from a single matching experiment including all three inoculums. B) Dose–response plot of MMV390048 control from validation assaysafter seeding HepG2 at 1.75 × 10^4^ cells/well and infecting with a sporozoite inoculum of 1.27 × 10^3^ for run 1 and 1.96 × 10^3^ for run 2. C) Dose–response plot of MMV390048 control from GHPB hit confirmation assaysafter seeding HepG2 at 5.00 × 10^3^ cells/well and infecting with a sporozoite inoculum of 1.22 × 10^3^ for run 1 and 1.33 × 10^3^ for run 2. Bars represent S.E.M. of replicate wells at each dose. [file 12936_2024_5155_MOESM2_ESM.jpg]

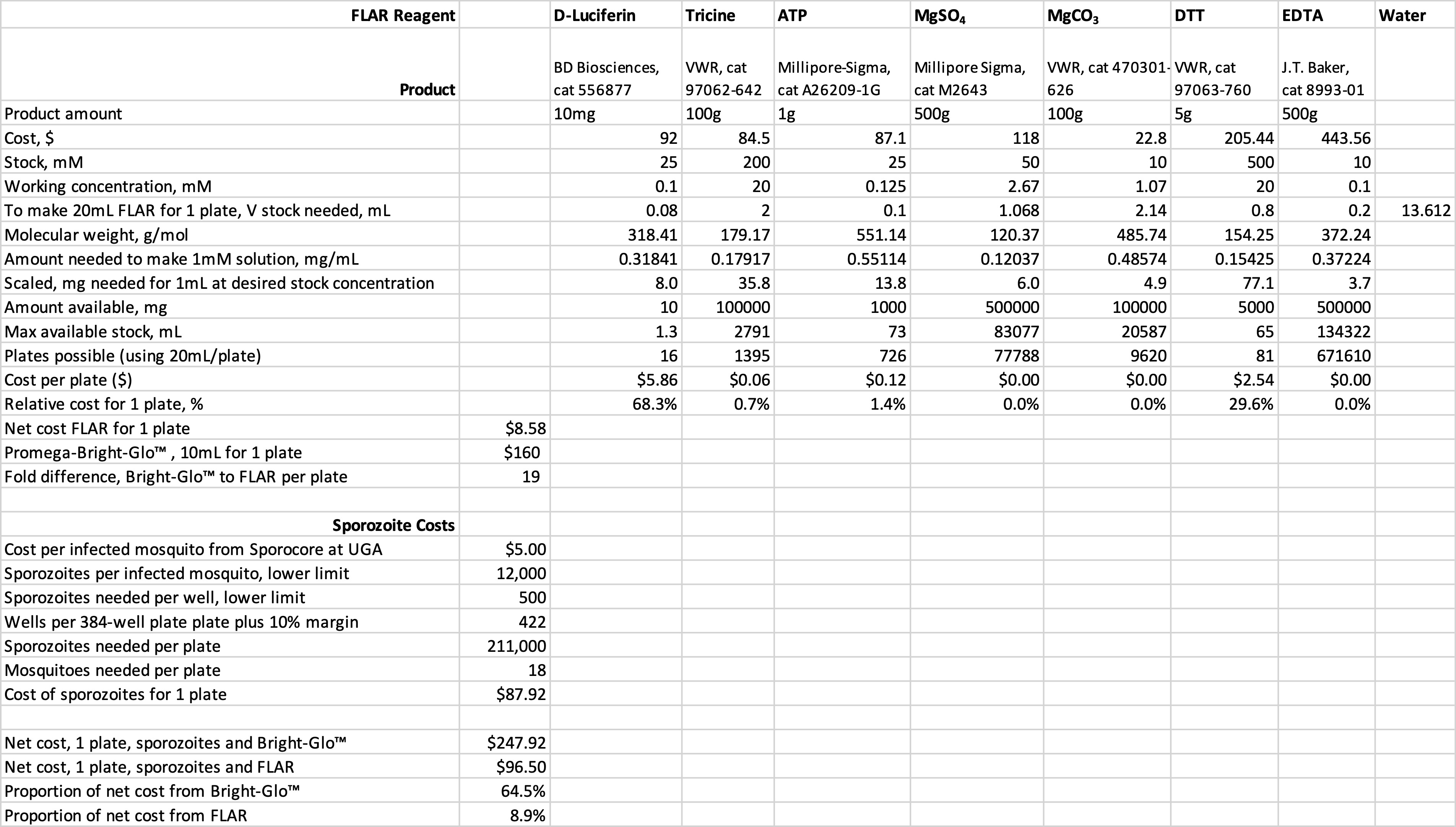

Supplement: Supplementary file 3 — Supplementary materials 3: Table 1. Cost analysis per plate for in-house FLAR reagent versus Bright-Glo™. [file 12936_2024_5155_MOESM3_ESM.jpg]
